# Supplementary material for: Immune Checkpoint Inhibitor, Nivolumab, Combined with Chemotherapy Improved the Survival of Unresectable Advanced and Metastatic Esophageal Squamous Cell Carcinoma: A Real-World Experience
Source: Int J Mol Sci. 2023 Apr 15;24(8):7312. doi: 10.3390/ijms24087312 (PMC10139434; doi:10.3390/ijms24087312)
Supplement: Supplementary file 1 [file ijms-24-07312-s001.zip › ijms-2310899-supplement/Figure S2.pdf]

(A) (B)

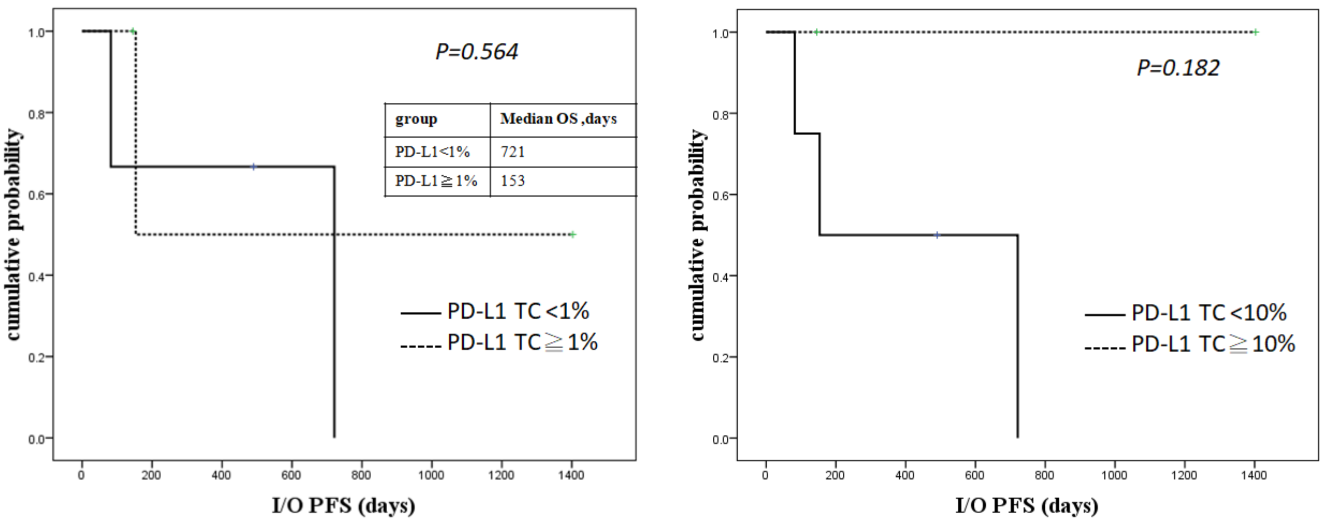

Figure S2 progression free survival (PFS) of patients received immunotherapy, including 5 nivolumab and chemotherapy and 1 dual immune check point inhibitor, on different PD-L1 tumor cells (TC) expression. (A) divided by PD-L1 TC <1% or ≧1%. (B)divided by PD-L1 TC <10% or ≧10%.
